# Research on the intervention of Mandala drawing therapy for social interaction disorders in children with autism

**DOI:** 10.3389/fpsyt.2026.1757575

**Published:** 2026-05-12

**Authors:** Lei Zhang, Ling Luo, Quanyuan Xia, Feifei Li

**Affiliations:** 1 School of Education, Zhaoqing University, Zhaoqing, China; 2 Yucai Third Kindergarten, Shenzhen, China; 3 College of Arts, Shandong Agricultural University, Taian, China

**Keywords:** Mandala drawing therapy, social interaction disorders, autism, children, ATEC

## Abstract

Social communication disorder is a core impairment in children with Autism Spectrum Disorder (ASD) and is essential for establishing connections with the external world. This study aimed to explore the effects of Mandala drawing therapy on social interaction and communication skills in children with ASD. Twenty-three children with ASD from H Special School were assigned to an experimental group or a control group. The experimental group received Mandala drawing therapy with standard rehabilitation training for one month, while the control group only participated in standard rehabilitation training. The Autism Treatment Evaluation Checklist (ATEC) was used to assess language, communication, and social skills before and after the intervention. Results showed that the experimental group exhibited greater within-group improvements in language, communication, and social skills compared with pre-test scores, whereas no statistically significant improvements were observed in the control group. No statistically significant between-group differences were detected post-intervention. These findings suggest that mandala drawing therapy, when used as an adjunct to standard rehabilitation, may contribute to within-group improvements in language and social communication skills, but no significant advantage over standard rehabilitation was observed.

## 1 Introduction

### 1.1 The growing challenge of autism spectrum disorder

According to the World Health Organization (WHO), the average prevalence of Autism Spectrum Disorder (ASD) among children globally is approximately 1% (1), which means ASD has emerged as a pressing concern. Current epidemiological data indicates a marked and consistent upward trend in the number of diagnosed cases of autism among children (2, 3). This rise has positioned ASD as one of the most prevalent neurodevelopmental conditions worldwide, surpassing other mental and developmental conditions in its rate of diagnosis. The implications of this trend extend far beyond individual health, imposing a substantial and multi-faceted burden on both society and families (4, 5). This burden encompasses direct medical and intervention costs, lost parental productivity, the emotional and psychological strain on caregivers, and the long-term societal costs associated with lifelong support and care (6). The increasing prevalence has galvanized both the public health sector and the academic community to seek a deeper understanding of the disorder and to develop more effective, accessible intervention strategies.

### 1.2 Core symptomatology and the primacy of social impairment

Autism Spectrum Disorder is characterized by a heterogeneous yet recognizable constellation of symptoms. The primary clinical manifestations, as outlined in diagnostic manuals such as the DSM-5, consistently encompass a triad of core challenges: persistent deficits in social interaction and social communication, a restricted range of interests, and the presence of repetitive and stereotyped behavior patterns (7, 8). These symptoms exist on a spectrum, leading to significant variability in the functional levels of individuals with ASD.

Among these core symptoms, the barrier to social interaction is arguably the most fundamental and debilitating. It constitutes the central axis around which many other challenges revolve (9–11). This social deficit is not merely a lack of interest but a profound difficulty in understanding and engaging in the reciprocal processes underlying social interaction. It manifests in challenges with non-verbal behaviors such as eye contact and body language, difficulties in developing peer relationships appropriate to developmental level, and a lack of spontaneous seeking to share enjoyment and interests with others. For a child with autism, the social world can appear as an indecipherable code, leading to frustration, anxiety, and behavioral withdrawal (12). Consequently, social skill, defined as the learned abilities that facilitate effective social interaction, play an essential role in enabling children to establish meaningful connections with others and are closely associated with their social integration, educational outcomes, and overall quality of life.

### 1.3 The critical window for intervention and the limits of current treatment

The neurodevelopmental nature of ASD underscores the critical importance of early diagnosis and intervention. The period of initial childhood years represents a phase of exceptional brain plasticity, characterized by rapid synaptogenesis and a heightened capacity for neural reorganization in response to environmental input (13). This neurological window of opportunity provides a unique timeframe during which targeted therapeutic interventions can be most effective in shaping neural pathways and establishing foundational social and communication skills. Early intervention during this period can help “wire” the brain for social engagement, potentially mitigating the severity of core symptoms and improving long-term developmental trajectories.

Despite this understanding, a significant challenge remains: there are currently no curative pharmaceutical drugs that address the core social and communicative symptoms of ASD. Pharmacological interventions may be used to manage co-occurring conditions such as anxiety, aggression, or hyperactivity, but they do not fundamentally alter the social deficit at the heart of autism. Therefore, the cornerstone of managing ASD lies in sustained, scientifically validated, and personalized non-pharmacological interventions. The search for effective psychosocial and behavioral interventions is not just an academic pursuit but a clinical imperative.

### 1.4 Mandala drawing therapy: a theoretical framework and its emergent evidence base

Within the expanding repertoire of non-pharmacological interventions, Mandala drawing therapy has garnered increasing attention as a promising art-based modality (14). Rooted in the analytical psychology of Carl Jung, Mandala drawing therapy is far more than a simple artistic activity. Jung perceived the mandala, a Sanskrit word meaning “circle,” as an archetypal symbol of wholeness and the Self, representing the innate striving of the psyche for integration and order (15, 16). In a therapeutic context, the creation of images within the structured, containing space of a circle provides a non-threatening framework for self-expression and internal organization.

For children, whose primary language is often play and symbolic expression, this modality holds particular appeal. It is essentially experienced as a structured game that aligns perfectly with children’s natural interests, thereby reducing resistance and fostering engagement (17). As a non-verbal medium, it offers a high-efficiency channel for information transmission, bypassing the language barriers that often confound traditional talk therapy for non-verbal or minimally verbal children with autism. It allows therapists and caregivers to gain a deeper and more empathetic understanding of the child’s inner world, including their emotions, conflicts, and potential, which the child may be unable to express verbally. This modality has been proposed to exert therapeutic effects for children with autism, including reducing anxiety, providing sensory integration, and facilitating emotional regulation (18).

A nascent but compelling body of empirical research has begun to substantiate these theoretical claims. Lu (19) adopted a controlled experimental design to verify that a specifically developed “Graffiti-Mandala drawing Intervention Model” could not only promote the development of drawing abilities in children with autism but, more importantly, enhance their broader psychological functions, suggesting a generalizable positive impact on their mental state. The application of Mandala therapy has also shown promise beyond ASD. For instance, Wang (20) conducted a study on 60 children with tic disorders, employing a combined approach of Mandala drawing therapy and sandplay therapy. The research found that this integrated method was effective in improving the children’s tic disorders, while also enhancing their self-awareness and general behavioral functioning, indicating its transdiagnostic potential for neurodevelopmental conditions. Collectively, these studies suggest that Mandala drawing therapy may yield positive effects in the holistic intervention for children with autism (21). This growing scientific recognition has been paralleled by a global increase in institutional and policy support for innovative autism rehabilitation. Governments and international bodies have continuously strengthened their commitment through laws, regulations, and the successive introduction of relevant policy documents, thereby providing a more robust infrastructure and guarantee for the treatment and education of children with autism.

### 1.5 Identifying the research gap and stating the aims of the present study

Despite these encouraging findings, a critical and specific research gap remains conspicuously absent from the literature. A systematic review of existing studies reveals that while Mandala therapy has been shown to improve general psychological functions, behavioral problems, and even fine motor skills, few studies have specifically examined a focused, empirical investigation specifically targeting its intervention effects on the core social disabilities of children with autism. While existing studies have examined related outcomes, the direct and targeted application of this modality to core social interaction deficits in autism remains limited. This gap represents a significant limitation in the field, as it prevents a comprehensive understanding of the full therapeutic potential of Mandala drawing therapy.

The present study aims to address this gap by exploring the potential role of structured Mandala drawing in supporting social development. It is hypothesized that the structured and contained nature of Mandala tasks may provide a non-threatening “relational space”, promoting engagement and social interaction in children with autism. This study adopts an empirical approach to investigate whether participation in a structured Mandala drawing therapy program is associated with improvements in social interaction abilities. The primary objectives of this research are as follows:

To implement a controlled, empirical trial comparing the social development of an experimental group receiving Mandala therapy against a control group.

To quantitatively compare and analyze the results of standardized assessments of social interaction abilities administered before and after the intervention period.

## 2 Methods

### 2.1 Participants

In the H Special School in Huizhou, China, 23 children aged 4–7 with autism were selected as subjects and divided into two groups. The experimental group consisted of 12 children (9 males and 3 females), while the control group had 11 children (8 males and 3 females). The sample size was determined by the number of eligible children available in the participating school during the study period. Due to the limited number of eligible participants, the final sample included 23 children. Accordingly, this study should be interpreted as a pilot feasibility study designed to explore preliminary effects rather than to establish definitive clinical efficacy. The study was approved by the Biomedical Ethics Committee of Huizhou University (Approval No.: HZUDIAC2408). Written informed consent was obtained from the parents or legal guardians of all participating children prior to participation. The study was conducted in a special education school as a school-based art intervention program.

### 2.2 Measures

#### 2.2.1 Autism Treatment Evaluation Checklist

This study centers on the social interaction challenges faced by children with autism, specifically focusing on the assessment of language and communication abilities, as well as social skills, through the “Autism Treatment Evaluation Checklist”, which comprises 34 items. When evaluating the dimensions of language communication and social interaction, a three-level scoring standard with reverse scoring is employed. For the assessment of social skills, a three-level scoring standard with positive scoring is utilized, where a lower score signifies a more favorable recovery status. This checklist is extensively utilized in the realm of autism research and diagnosis, exhibiting robust reliability and validity (22).

#### 2.2.2 The “Datura Growth and Self-Healing Picture Book” album

**Figure 1:**
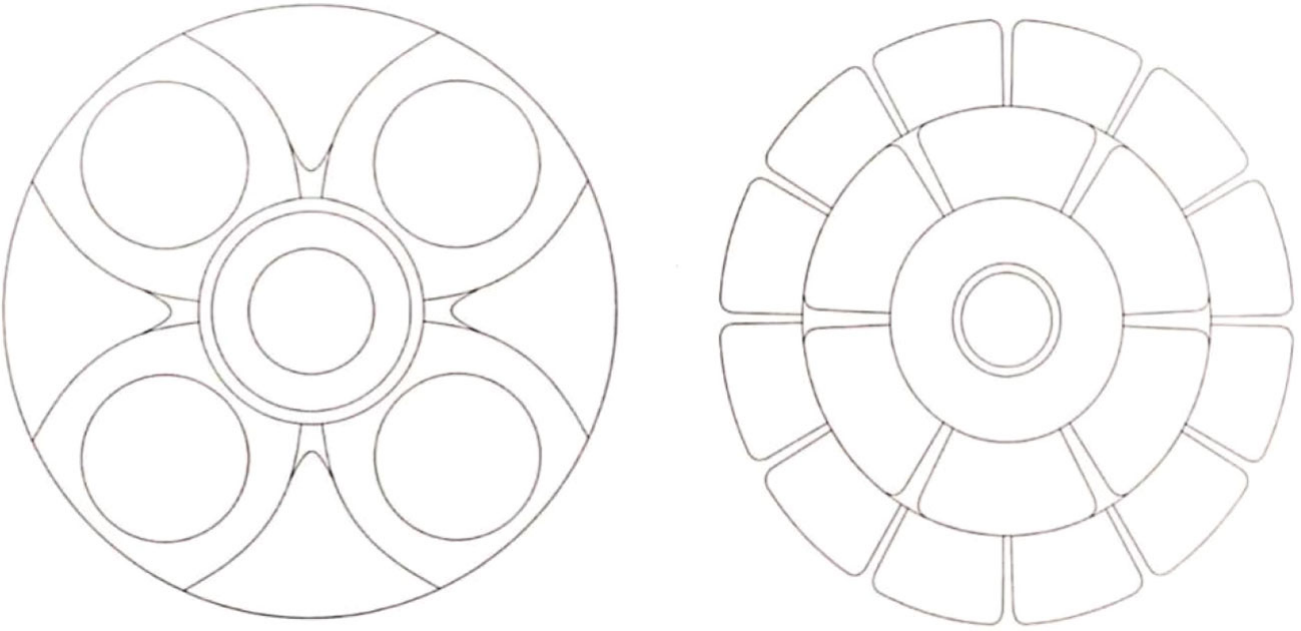
Mandala drawing graphics.

This album was developed by Dr. Chen and Dr. Gao based on their long-term psychological research and counseling experience, specifically tailored for the psychological characteristics and common issues of the Chinese population (23). Considering the special circumstances of children with autism, this study selected 8 images from the album for children with autism to color in order of increasing difficulty, as shown in **Figure 1**. The eight drawing tasks were selected based on developmental appropriateness and progressively increasing complexity, taking into account the cognitive and motor abilities of children aged 4–7 years with Autism Spectrum Disorder. These criteria ensure that tasks were engaging, achievable, and gradually challenged participants’ drawing and attention skills.

#### 2.2.3 Observation record table

This study primarily utilized unstructured observation methods, directly observing the behaviors of children with autism during mandala drawing group assistance activities. The observation results were flexibly recorded in accordance with actual conditions, as illustrated in **Table 1**.

**Table 1:** Observation record of Mandala drawing group counseling activities for kindergarten class at H Special School.

Observed class: Observer: Observation target: Gender: Observation purpose: Observe the behavior and artistic works of the children in the experimental group during the mandala drawing group counseling activities.		
Observation date	Drawing works	Behavioral performance

### 2.3 Treatment

#### 2.3.1 Research design

Group allocation was conducted at the class level rather than the individual level to minimize potential contamination between participants. Children within the same class interact frequently during daily school activities, including lessons, playtime, and group exercises. Randomly assigning individual children from the same class to different groups could have resulted in the sharing of intervention content between the experimental and control groups, thereby reducing the ability to isolate the effects of the mandala drawing therapy. By assigning entire classes to a single study group, all children within a class received the same intervention, ensuring that observed differences between groups could be attributed to the intervention while maintaining the integrity of daily classroom interactions.

Two classes was randomly selected as the control group and experimental group. Then, 23 autistic children from H Special School were divided into a control group of 11 and an experimental group of 12. Participants in the control group received conventional rehabilitation training, which consisted of classroom-based activities focusing on language skills, social interaction, and basic cognitive exercises. Sessions were conducted by the same teachers as in the experimental group and followed the same schedule and duration to maintain consistency. No mandala drawing activities were included, ensuring that the intervention variable was isolated in the experimental group. While the experimental group also underwent Mandala drawing Therapy intervention, in a group setting, 2–3 times a week, each session lasting 40 minutes, for a total of 9 sessions over 1 month, using the patterns from the “Mandala Growth Self-Healing Picture Book” (23), adhering to the principle of “people-oriented” to help improve their social communication disorders.

#### 2.3.2 Research process

In the experiment, the experimental group’s rehabilitation training included conventional teaching and mandala drawing group counseling, while the control group only had conventional teaching. The mandala drawing therapy intervention was the variable. Intervention fidelity was monitored and ensured through a standardized protocol. The group counseling sessions were delivered by the same facilitator using consistent content and procedures. The facilitator received structured training prior to implementation, and ongoing supervision was conducted throughout the study to monitor consistency. During the experiment, both groups were prohibited from participating in other rehabilitation treatments and taking medication on their own to ensure that the therapy intervention was the only variable.

##### 2.3.2.1 Assessment before the experimental intervention

Before the initiation of the experimental study, the classroom teachers conducted an assessment of the autism children in the experimental and control groups using the Autism Treatment Evaluation Checklist (ATEC). The assessment primarily focused on two key dimensions: language communication and social interaction skills.

##### 2.3.2.2 Group counseling activities during intervention

The intervention was conducted as a group counseling program. A trained facilitator led 12 children with autism in the experimental group through Mandala coloring activities and structured experience sharing. Each session lasted 40 minutes and consisted of three phases: a 2-minute activity introduction, a 30-minute drawing activity, and an 8-minute sharing and feedback session. During the introduction, familiar and easy-to-follow rhythmic cues were used to engage the children and create a relaxed atmosphere. In the drawing phase, structured Mandala templates were provided. The facilitator demonstrated basic coloring techniques and gave simple, structured instructions, after which participants were encouraged to select colors independently and engage in spontaneous creation. Assistants offered individualized support and guidance as needed. A total of eight Mandala tasks were used, systematically categorized into levels of difficulty based on visual complexity, pattern density, and required fine motor skills, progressing from simple to more complex designs. In the feedback phase, the facilitator provided positive evaluations and encouraged children’s efforts and creativity.

##### 2.3.2.3 Evaluation after experimental intervention

After completing the mandala drawing therapy activities, the researchers used the same scales and methods as the initial assessment to reassess the children in the experimental and control groups, collected the data, and conducted a comparative data analysis of the overall condition of the children.

## 3 Results

### 3.1 Comparison of pre-test results on language and communication, social skills of the ATEC between the control group and the experimental group

Social interaction skills in this study were assessed using the language and communication and social skills subscales of the Autism Treatment Evaluation Checklist (ATEC). Prior to statistical analysis, the normality of the pre-test data was examined using the Shapiro-Wilk test. The null hypothesis (H0) assumed that the sampled data were drawn from a normally distributed population. The results are presented in **Table 2**. The analysis indicated that all pre-test variables followed a normal distribution, except for the social skills scores of the control group.

**Table 2:** Normality test results of pre-test data.

Shapiro-wilk test		Statistic	df	Sig.
Language and communication	Control group	0.893	11	0.150
	Experimental group	0.939	12	0.484
Social skills	Control group	0.833	11	0.026*
	Experimental group	0.935	12	0.437

*.P<0.05, indicating a significant difference.

Independent-samples t-tests were conducted to compare the scores on the language and communication dimension between the two groups. First, the homogeneity of variance was examined using Levene’s test. The results showed that F = 15.445 and p<0.001, indicating that the assumption of equal variances was violated. Therefore, Welch’s t-test was applied. The results of the pre-test comparison between the control group and the experimental group are presented in **Table 3**. The analysis of the pre-intervention data indicated that there was no statistically significant difference in language and communication scores between the two groups.

**Table 3:** Comparison of pre-test results of language and communication dimension between experimental group (ATEC) (N = 23).

Variable	Control group( $\bar{\text{x}}$ ± s)	Experimental group ( $\bar{\text{x}}$ ± s)	Mean difference	t	P	Cohen’s d
Language and communication	15.91 ± 7.569	15.33 ± 3.312	-0.576	-0.233	0.820	0.1

The Mann-Whitney U test was employed to examine the differences between the control group and the experimental group in the social dimension, with the results presented in **Table 4**. The results indicated no significant differences in the pre-test data of the social dimension between the control group and the experimental group.

**Table 4:** Comparison of pre-test results of social skills dimension (ATEC) between control group and experimental group (N = 23).

Variable	H0	Test	Sig.	Decision
Social skills	There was no significant difference in the pre-test data of the social dimension between the control group and the experimental group.	Mann-Whitney U test	0.880	Fail to reject H_0_

Therefore, it can be concluded that before the experimental intervention, there were no significant differences in the development of language and communication, and social skills between the children in the control group and the experimental group. The absence of statistically significant differences at baseline indicates that the experimental and control groups were comparable prior to the intervention. Additionally, the ages of the children in both groups were similar, and they only participated in rehabilitation training activities at school, which resulted in little difference in the levels of abilities presented during the assessment.

### 3.2 Comparison of post-test results on the language and communication, social skills of the ATEC between the control group and the experimental group

First, the normality of the post-test data was assessed using the Shapiro-Wilk test. The null hypothesis (H0) assumed that the data were drawn from a normally distributed population. As presented in **Table 5**, the post-test scores for the language dimension met the assumption of normality, whereas the social skills scores did not follow a normal distribution.

**Table 5:** Normality test results of post-test data.

Shapiro-Wilk test		Statistic	df	Sig.
Language and communication	Control group	0.901	11	0.190
	Experimental group	0.954	12	0.697
Social skills	Control group	0.822	11	0.018*
	Experimental group	0.835	12	0.024*

*.P<0.05, indicating a significant difference.

Based on the scores of the samples, an independent samples T-test was conducted on language and communication dimension. First, the homogeneity of variance was tested, with F = 10.372, P = 0.004<0.05, indicating non-homogeneous variance. Therefore, the corrected t-test (Welch method) was employed and the statistical results of the post-test for the control group and the experimental group are presented in **Table 6**. The post-test analysis showed no significant differences in language and communication scores between the experimental and control groups.

**Table 6:** Comparison of post-test results for the language and communication, social skills in the ATEC between the control group and the experimental group (N = 23).

Variable	Control group ( $\bar{\text{x}}$ ± s)	Experimental group( $\bar{\text{x}}$ ± s)	Mean difference	t	P	Cohen’s d
Language and communication	16.09 ± 8.031	13.25 ± 4.070	-2.841	-1.056	0.308	0.453

The Mann-Whitney U test was employed to analyze the differences between the control group and the experimental group in the social dimension, with the results presented in **Table 7**. The findings indicated no significant differences in the post-test data of the social dimension between the two groups.

**Table 7:** Comparison of pre-test results of social skills between control group and experimental group using the ATEC (N = 23).

Variable	H0	Test	Sig.	Decision
Social skills	There was no significant difference in the post-test data of the social dimension between the control group and the experimental group.	Mann-Whitney U test	0.169	Fail to reject H_0_

### 3.3 The comparison of pre- and post-test results of the experimental group on the ATEC scale for language and communication, social skills

According to the results of the normality test, the pre- and post-test data for the language and communication dimension in the experimental group followed a normal distribution. Therefore, a paired-sample t-test was conducted. The comparison results between the pre-test and post-test for the experimental group are presented in **Table 8**. Before the intervention, the mean score of the experimental group on the language and communication subscale of the Autism Treatment Evaluation Checklist (ATEC) was 15.33 ± 3.312. After the intervention, the mean score decreased to 13.25 ± 4.070. The results indicated a statistically significant difference in the language and communication scores of the experimental group before and after the intervention (p < 0.05). Effect size (Cohen’s d = 1.080) values suggest a large magnitude of change in the experimental group.

**Table 8:** Comparison of pre- and post-test results for the ATEC scale in language and communication of the experimental group (N = 12).

Variable	Control group ( $\bar{\text{x}}$ ± s)	Experimental group( $\bar{\text{x}}$ ± s)	Mean difference	t	P	Cohen’s d
Language and communication	15.33 ± 3.312	13.25 ± 4.070	-2.08	3.742	0.003*	1.080

*.P<0.05, indicating a significant difference;.

Based on the results of the normality test, the post-test data for the social dimension in the experimental group did not follow a normal distribution. Therefore, the Wilcoxon signed-rank test was used to analyze the differences between the pre-test and post-test scores in the social dimension for the experimental group. The results are presented in **Table 9**. The findings indicate a statistically significant difference between the pre-test and post-test scores for the social dimension in the experimental group.

**Table 9:** Comparison of pre- and post-test results for social skills dimension (ATEC) in the experimental group (N = 12).

Variable	H0	Test	Sig.	Decision
Social skills	There was no significant difference between the pre-test and post-test scores for the social dimension in the experimental group.	Wilcoxon signed-rank test	0.002*	reject H_0_

*.P<0.05, indicating a significant difference.

### 3.4 The comparison of pre- and post-test results of the ATTEC scale language and communication, social skills in the control group

Based on the results of the normality test, the pre-test and post-test data for the language and communication dimension in the experimental group followed a normal distribution. Therefore, a paired-sample t-test was conducted. The statistical results of the pre-test and post-test of the control group was shown in **Table 10**. There was no significant difference in the pre-test and post-test results of language and communication (ATEC) for the control group (p >.05).

**Table 10:** Comparison of pre- and post-test results for the ATEC scale language and communication in the control group (N = 11).

Variable	Pre-test ( $\bar{\text{x}}$ ± s)	Post-test ( $\bar{\text{x}}$ ± s)	Mean difference	t	P	Cohen’s d
Language and communication	15.91 ± 7.569	16.0 ± 8.031	0.180	-0.239	0.816	-0.072

According to the results of the normality test, the pre-test and post-test data for the social skills dimension in the control group did not follow a normal distribution. Therefore, the Wilcoxon signed-rank test was used to examine the differences between the pre-test and post-test scores for the social skills dimension in the control group. The results are presented in **Table 11**. The findings indicated that there was no statistically significant difference between the pre-test and post-test scores for social skills in the control group.

**Table 11:** Comparison of pre- and post-test results for the ATEC scale social skills in the experimental group (N = 12).

Variable	H0	Test	Sig.	Decision
Social skills	There was no significant difference between the pre-test and post-test scores for social skills dimension in the control group.	Wilcoxon signed-rank test	0.181	Fail to reject H_0_

## 4 Discussion

### 4.1 Experimental outcomes and statistical analysis

The present study yielded significant findings regarding the efficacy of Mandala drawing therapy as an intervention for children with Autism Spectrum Disorder (ASD). A thorough analysis of baseline measurements confirmed the fundamental prerequisite for valid experimental comparison: there were no statistically significant differences in the initial scores between the control group and the experimental group across the key domains of language and communication, as well as sociability. This initial equivalence (p>0.05) establishes that both groups were comparable in their developmental profiles prior to the intervention, thereby strengthening the validity of subsequent comparisons and allowing for more confident attribution of any observed changes to the experimental manipulation.

Following the implementation of the Mandala drawing therapy intervention for the experimental group, a comprehensive paired-samples statistical analysis revealed compelling within-group changes. The post-test scores for the experimental group demonstrated statistically significant differences from their pre-test scores in both language and communication (p<0.05) and sociability (p<0.05). This significant divergence between pre- and post-intervention measurements within the same group provides strong preliminary evidence that the Mandala drawing therapy had a measurable and positive therapeutic effect on these core developmental areas for the children with autism (24, 25).

A crucial aspect of the experimental design was the control of concurrent interventions. Throughout the study period, both the experimental and control groups continued to participate in the standard rehabilitation curriculum provided by the special education school. This program typically included speech therapy, occupational therapy, and behavioral interventions, which represent the standard form of care in the institution. Despite this shared background of ongoing therapeutic input, the pre-test and post-test results for the control group showed no statistically significant differences in either language/communication or sociability domains (p>0.05 for both within-group comparisons). In contrast, the experimental group demonstrated significant improvements after the intervention. This difference in outcomes suggests that conventional rehabilitation training alone may help maintain existing skills or produce gradual progress, but it may not lead to the same degree of measurable short-term improvement as the additional Mandala drawing therapy. Therefore, the significant improvements observed in the experimental group indicate that the enhancement in language, communication, and social interaction skills may be attributed to the Mandala drawing intervention, which likely functioned as a complementary component to the standard rehabilitation program.

### 4.2 Nuanced findings and methodological considerations

A more nuanced finding emerged from the between-group comparison of post-test scores. Although the experimental group demonstrated lower mean post-test scores (indicating improvement on the ATEC scale) than the control group in both language/communication and social skills dimensions, these between-group differences did not reach statistical significance at the post-intervention stage. This discrepancy between significant within-group improvements and a non-significant between-group difference warrants careful interpretation.

The most plausible explanation for this outcome lies in the methodological limitation of the present study, particularly the relatively small sample size. With only 23 participants in total, divided into two groups, the statistical power of the analysis was inherently limited. Statistical power refers to the probability that a statistical test will correctly reject a false null hypothesis (i.e., detect a true effect when one truly exists). When statistical power is low, the likelihood of committing a Type II error increases, meaning that a real effect may exist but fails to reach statistical significance due to limited data and high variability. In the present study, although the experimental group consistently showed better performance trends than the control group, these differences may not have reached statistical significance in direct between-group comparisons because of the limited sample size. The restricted sample size, combined with variability in participant characteristics, may therefore have obscured potentially meaningful intervention effects. In addition, the relatively large standard deviation observed in the pre-test social skills scores of the control group indicates considerable heterogeneity among participants. Such variability is common among children with Autism Spectrum Disorder within the same age range, as individual differences in social communication abilities can be substantial. This heterogeneity may further influence the stability and sensitivity of statistical comparisons.

Therefore, although the findings suggest that Mandala drawing therapy may have beneficial effects, the results should be interpreted with caution given the relatively small sample size, the variability observed in baseline social skills within the control group, and the inherent heterogeneity of children with Autism Spectrum Disorder. Future studies with larger samples are needed to further evaluate the effectiveness of Mandala drawing therapy compared with standard rehabilitation alone and to obtain more precise estimates of the intervention effect.

### 4.3 Consistency with existing literature and theoretical framing

Notwithstanding this methodological consideration, the core finding of significant within-group improvement for the experimental group aligns robustly with a growing body of international scholarly work. The results resonate strongly with the findings of Lu et al. (26), who independently concluded that a structured Mandala drawing intervention could actively promote the development of language and social abilities in children with autism by providing a structured outlet for non-verbal expression and emotional regulation. Similarly, the research results of Zhou and Liu (27) demonstrated that integrating Mandala drawing therapy into a comprehensive treatment plan could synergistically enhance the overall effectiveness of rehabilitation, yielding specific and measurable gains in language, communication, and sociability beyond what was achieved through conventional methods alone. More recently, the work of Liu et al. (28) provided further corroboration, indicating through a longitudinal design that a sustained Mandala drawing therapy intervention could effectively strengthen these very skills in autistic children, with effects maintained at a one-month follow-up assessment. The convergence of these findings across multiple independent studies, employing slightly different methodologies but focusing on the same core intervention, significantly bolsters the validity of the present results and points to the reliability of Mandala therapy as a beneficial adjunctive intervention for ASD.

The mechanisms underlying these improvements can be understood through a multi-faceted theoretical lens, combining social learning theory with sensory integration and psychoanalytic frameworks. The structured, group-based nature of Mandala activities inherently creates a scaffolded environment for social learning. Unlike unstructured free play, which can be overwhelming for children with ASD, the Mandala circle provides a predictable and bounded space for interaction. These sessions provide invaluable, low-pressure opportunities for children to engage in proto-social behaviors with both peers and instructors. The acts of sharing their completed works, offering simple comments on the creations of others, navigating the shared use of art materials, and simply engaging in a parallel, focused activity in a communal setting all necessitate and foster basic communication and a nascent understanding of others’ perspectives and feelings (29, 30).

During the experimental sessions, these theoretical benefits were translated into observable, quantifiable behavioral changes. Researchers maintained detailed ethnographic field notes that documented a clear evolution in social engagement. Children in the experimental group began to demonstrate more proactive and initiated social behaviors, such as offering spontaneous, context-appropriate greetings (e.g., “Hello, teacher”), verbally identifying colors used in their artwork (“I use blue”), and more readily responding to direct and open-ended questions. It appeared that within the familiar, predictable, and aesthetically structured context of the Mandala drawing environment, the children felt a greater sense of psychological safety. This security, born from the ritualistic and repetitive nature of the activity, in turn lowered their ambient anxiety and increased their intrinsic motivation to explore and participate in the social microcosm of the group, thereby organically increasing their overall social engagement frequency and duration.

### 4.4 Observed behavioral and emotional benefits

Beyond the quantitatively measured metrics of language and social skills, the intervention yielded profound positive effects on the children’s emotional well-being, attentional capacity, and overall engagement. The researchers observed that the children in the experimental group began to eagerly anticipate the group counseling sessions, with many demonstrating behaviors indicative of anticipation and positive expectation. The atmosphere within these sessions evolved from one of tentative participation to progressively more lively, positive, and mutually responsive. A particularly noteworthy observation was that some children demonstrated a qualitatively and quantitatively superior level of sustained concentration and focus during Mandala drawing sessions compared to their engagement in other classroom or therapy activities. They often worked in a visibly cheerful and absorbed mood, occasionally erupting in spontaneous, genuine laughter while selecting colors or filling in patterns—a behavior rarely observed in their other routines.

This subjective impression of heightened engagement was powerfully corroborated by objective, third-party anecdotal evidence from the children’s primary caregivers. Their class teacher, who was blind to the specific hypotheses of the study but observed the children daily, offered a poignant unsolicited comment that powerfully illustrates the motivational pull and emotional significance the Mandala sessions held for the children. The teacher stated, “Before the group counseling activities, some children would often look out the window, waiting for your arrival. On Mandala days, they were noticeably more attentive to the passage of time and needed fewer prompts to transition to the activity.” This statement serves as a powerful testament to the intrinsic reward value and motivational salience that the intervention held for the participants. These observations provide qualitative insights into participant engagement but should be interpreted as descriptive rather than quantitative evidence.

### 4.5 Underlying therapeutic mechanisms

The therapeutic power of Mandala drawing for the ASD population can be attributed to a confluence of its unique structural and symbolic characteristics. Firstly, it provides a safe, primarily non-verbal, and visually-mediated avenue for self-expression, which is critically important for a population characterized by pervasive communication deficits. The activity is inherently captivating, attracting and holding visual attention through its symmetry and pattern, which in turn helps to reduce underlying anxiety and sensory overwhelm by providing a focused point of reference (17). By channeling cognitive resources into the structured, sensory-rich, and goal-oriented task of coloring within the mandala’s predefined forms, children can experience a “flow state”, temporarily disengaging from external distractions and internal pressures, and entering a state of relative calm and mental focus that is neurologically regulating. Studies in neuroaesthetics have consistently shown that structured artistic activities like mandala coloring can modulate brainwave patterns, increasing alpha wave activity associated with relaxed alertness, which is particularly beneficial for children with ASD who often experience heightened arousal and anxiety (19).

Moreover, mandala drawing offers distinct therapeutic advantages over general free-form drawing or other art activities (31). Its defining features, including the concentric circle, radial symmetry, and central point, resonate deeply with the psychological and neurological preference many autistic children have for predictability, order, and visual clarity. The absence of ambiguous, open-ended space reduces the cognitive load and anxiety associated with unstructured creativity. The outer circle of the mandala is not just a physical boundary; it is often perceived and experienced symbolically as a protective container, a “temenos” or sacred space that safely holds psychic content (32). This structure subconsciously provides a powerful sense of security, containment, and warmth, creating a non-threatening “holding environment” for the psyche where the child can explore their inner world without fear of fragmentation or overwhelm.

Therefore, Mandala psychological drawing, by its very structure, aligns with the intrinsic psychological characteristics and needs of children with autism. It functions not merely as an art project, but as a powerful, non-threatening medium through which they can process their experiences, regulate their emotions, and initiate communication with the external world. It offers a unique, evidence-based blend of structural safety and expressive freedom that effectively meets the children at their point of need, facilitating growth in core deficit areas through a modality they find intrinsically rewarding and accessible. The findings support the potential value of Mandala drawing therapy as a complementary intervention and provide a foundation for future controlled studies evaluating its clinical effectiveness. However, these findings should be interpreted as preliminary and exploratory, highlighting the need for larger controlled studies to confirm the observed trends.

## 5 Conclusion and limitation

This study explored the potential effects of Mandala drawing therapy as a complementary intervention for children with Autism Spectrum Disorder in a special education setting. The results showed significant within-group improvements in language and social communication skills in the experimental group following the intervention. However, no statistically significant differences were observed between the experimental and control groups after the intervention. These findings suggest that mandala drawing therapy, when used as an adjunct to standard rehabilitation, may contribute to within-group improvements in language and social communication skills, but no significant advantage over standard rehabilitation was observed.

Given the relatively small sample size and the heterogeneity of participants, the findings should be interpreted with caution. Future research should involve larger-scale randomized controlled trials to further evaluate the effectiveness of Mandala drawing therapy, investigate the long-term maintenance of gains, and explore the underlying neural mechanisms of change using neuroimaging techniques, thereby clarifying its potential role in autism rehabilitation programs.

## Data Availability

The raw data supporting the conclusions of this article will be made available by the authors, without undue reservation.
